# Long-term associations between early-life family functioning and preadolescent white matter microstructure

**DOI:** 10.1017/S0033291722001404

**Published:** 2023-07

**Authors:** Scott W. Delaney, Yllza Xerxa, Ryan L. Muetzel, Tonya White, Sebastien Haneuse, Kerry J. Ressler, Henning Tiemeier, Laura D. Kubzansky

**Affiliations:** 1 Department of Social and Behavioral Sciences, Harvard T.H. Chan School of Public Health, Boston, MA USA; 2 Department of Child and Adolescent Psychiatry, Erasmus University Medical Center, Rotterdam, the Netherlands; 3 Lee Kum Sheung Center for Health and Happiness, Harvard T.H. Chan School of Public Health, Boston, MA USA; 4 The Generation R Study Group, Erasmus University Medical Center, Rotterdam, the Netherlands; 5 Department of Epidemiology, Erasmus University Medical Center, Rotterdam, the Netherlands; 6 Department of Radiology and Nuclear Medicine, Erasmus University Medical Center, Rotterdam, the Netherlands; 7 Department of Biostatistics, Harvard T.H. Chan School of Public Health, Boston, MA USA; 8 Department of Psychiatry, McLean Hospital, Harvard Medical School, Belmont, MA USA

**Keywords:** Child development, Family functioning, Cocial environment, White matter

## Abstract

**Background:**

Causes of childhood behavior problems remain poorly understood. Enriched family environments and corresponding brain development may reduce the risk of their onset, but research investigating white matter neurodevelopmental pathways explaining associations between the family environment and behavior remains limited. We hypothesized that more positive prenatal and mid-childhood family functioning – a measure of a family's problem solving and supportive capacity – would be associated with two markers of preadolescent white matter neurodevelopment related to reduced behavior problems: higher global fractional anisotropy (FA) and lower global mean diffusivity (MD).

**Methods:**

Data are from 2727 families in the Generation R Study, the Netherlands. Mothers reported family functioning (McMaster Family Assessment Device, range 1–4, higher scores indicate healthier functioning) prenatally and in mid-childhood (mean age 6.1 years). In preadolescence (mean age 10.1), the study collected diffusion-weighted scans. We computed standardized global MD and FA values by averaging metrics from 27 white matter tracts, and we fit linear models adjusting for possible confounders to examine global and tract-specific outcomes.

**Results:**

Prenatal and mid-childhood family functioning scores were moderately correlated, *r* = 0.38. However, only prenatal family functioning – and not mid-childhood functioning – was associated with higher global FA and lower global MD in preadolescence in fully adjusted models: *β*_global FA_ = 0.11 (95% CI 0.00, 0.21) and *β*_global MD_ = −0.15 (95% CI −0.28, −0.03) per one-unit increase in functioning score. Sensitivity and tract-specific analyses supported these global findings.

**Conclusions:**

These results suggest high-functioning prenatal or perinatal family environments may confer lasting white matter neurodevelopmental benefits into preadolescence.

## Introduction

The origins of child behavior disorders remain poorly understood. Increasingly, investigators have called for a population neuroscience approach both to identify factors shaping brain development, and to understand how variations in brain development cause behavior problems (Paus, 2010; Tiemeier & Muetzel, 2020). Research suggests that both positive and negative aspects of the social environment impact brain development in ways that may affect behavior problems (Liu, 2004; Plybon & Kliewer, 2001). These aspects include experiences related to one's family, friends, school, neighborhood, and place of worship (Bronfenbrenner, 1986). The relative importance of these domains may change throughout childhood, with the family environment most influential early in life before children develop relationships outside their home. As such, a healthy early-life family environment may drive healthy brain development and protect against behavior disorders.

However, the neurodevelopmental effects of family-based exposures have not been thoroughly explored. Among studies in this area, most focus on family dysfunction and its link to poor outcomes (Xerxa et al., 2021). For example, a broad body of research links child maltreatment, which often occurs within the family, to brain structure alterations related to behavior problems (Teicher, Samson, Anderson, & Ohashi, 2016). Similarly, functional imaging studies report that family conflict is associated with increased adolescent risk-taking behavior (Guassi Moreira & Telzer, 2018; McCormick, Qu, & Telzer, 2016).

In contrast to family dysfunction research, some neurodevelopmental studies investigate positive family-based experiences, which may confer benefits beyond those associated with a mere absence of negative experiences (Bhanot, Bray, McGirr, Lee, & Kopala-Sibley, 2021). For example, greater maternal support and positive parenting behavior have been associated with brain changes thought to be advantageous, including accelerated hippocampal growth in childhood and adolescence, and attenuated amygdala growth in adolescence (Luby, Belden, Harms, Tillman, & Barch, 2016; Whittle et al., 2014). Some functional imaging studies also report associations between healthy parent–child relationships, decreased risk-taking behavior, and increased cognitive control in adolescence and early adulthood (Holmes et al., 2018; Kim-Spoon, Maciejewski, Lee, Deater-Deckard, & King-Casas, 2017; Qu, Fuligni, Galvan, & Telzer, 2015).

These studies, however, focus on parenting practices rather than on broader measures of overall family functioning that may capture different characteristics within a complex family ecology. Many of these studies also assess exposures during a narrow time period in a child's life. As a result, they do not aim to quantify how the family environment's influence may change throughout childhood. And despite the importance of white matter to healthy brain development, prior family environment imaging studies assess only functional or gray matter structural outcomes.

Both negative and positive experiences occurring prenatally and in childhood likely alter white matter development (Lebel & Deoni, 2018; Paus, Pesaresi, & French, 2014). Studies report associations between negative exposures (e.g. maternal prenatal anxiety) and properties of white matter microstructure that may decrease neural efficiency, and associations between positive exposures (e.g. breastfeeding) and the opposite (Bick et al., 2015; Deoni et al., 2013; Hanson, Knodt, Brigidi, & Hariri, 2015; Jensen et al., 2018; Rifkin-Graboi et al., 2015). These studies are complemented by a separate body of research associating white matter microstructure with behavioral outcomes. Specifically, microstructural properties related to more efficient neural processing (e.g. higher fractional anisotropy (FA) and lower mean diffusivity (MD), discussed in more detail below) are generally associated with fewer behavior problems, while microstructural properties related to less efficient neural processing are associated with disruptive behavior problems and psychopathology (Bolhuis et al., 2019; Lebel & Deoni, 2018; Waller, Dotterer, Murray, Maxwell, & Hyde, 2017).

To investigate whether a positive family environment may impact white matter microstructure, we used prospective data from the Generation R Study, a large, population-based birth cohort. Researchers collected data on family functioning from mothers prenatally and in mid-childhood, and their children completed an MRI brain scan in preadolescence. We hypothesized that more positive family functioning at each timepoint would be associated with more organized white matter microstructure across all areas of the brain (i.e. global effects), even after extensive adjustment for plausible confounders selected based on prior literature and theory (Lebel & Deoni, 2018).

## Methods

### Participants

We used data from the Generation R Study, a population-based birth cohort in Rotterdam, the Netherlands, seeking to identify factors affecting healthy child development (Kooijman et al., 2016). The study enrolled 9778 women during pregnancy or shortly after giving birth who were living in Rotterdam between 2002 and 2006. Researchers have collected data from children and their caregivers at multiple timepoints through the present after securing participants' written informed consent and assent. All study protocols are approved by the Medical Ethics Committee of the Erasmus University Medical Center.

Women completed a postal questionnaire about their family functioning prenatally (gestational age 18–25 weeks) and again when their child was in mid-childhood (mean age 6.0 years; range 4.0–9.1 years). Mothers enrolled at the birth of their child (i.e. not while pregnant) completed only the mid-childhood questionnaire. 8234 mothers completed at least one of these questionnaires. Later, researchers scanned preadolescent children (mean age 10.1 years; range 8.6–12.0 years) with diffusion-weighted magnetic resonance imaging (DWI) (White et al., 2018). The current study included participants with a usable DWI scan (described below) and either prenatal or mid-childhood family functioning data (*n* = 2914). Among these participants, we excluded those exposed in utero to cocaine or heroin (*n* = 8). Because Generation R includes a number of twins and triplets, we randomly selected only one sibling for inclusion in these cases (*n* = 37 removed). We also excluded extremely low birthweight babies (birthweight < 1000 g; *n* = 5) given the confounding complexity of their postnatal care. After removing participants with any missing tract-specific scalar data (*n* = 55) or outlying values (described below, *n* = 82), our final analytic sample included 2727 children. Online Supplementary Section 1 details selection into our analytic sample.

### Measures

#### Family functioning

To measure family functioning, mothers completed the McMaster Family Assessment Device, General Functioning Subscale via a postal questionnaire. This is a 12-item self-report survey of established reliability and validity in Dutch and several other populations, in which mothers respond on a four-point Likert scale to 6 positively-framed and 6 negatively-framed items (Boterhoven de Haan, Hafekost, Lawrence, Sawyer, & Zubrick, 2015; Byles, Byrne, Boyle, & Offord, 1988; Epstein, Baldwin, & Bishop, 1983; Wenniger, Hageman, & Arrindell, 1993). Representative questions include, ‘If there are problems, we can count on each other for support’, and, ‘There are a lot of unpleasant and painful feelings in our family’. Because these questions do not reference specific family members or roles, mothers can respond regardless of their family's structure. We averaged responses to all items, reverse scoring where necessary, to derive a continuous family functioning score (range 1–4), in which higher scores indicate better functioning. Scores around the range's midpoint of 2.5 indicate levels of family functioning that are neither highly negative nor highly positive, while scores considerably above the midpoint are attainable only if mothers report the presence of positive family functioning and the absence of negative functioning. Cronbach's alpha in the analytic sample was strong (0.89) at both prenatal and mid-childhood time periods.

#### Brain imaging

Generation R researchers have described diffusion-weighted imaging protocols and processing elsewhere (Muetzel et al., 2018; White et al., 2018). All DWI images were acquired using a 3T GE MR-750W scanner (General Electric, Milwaukee, Wisconsin) and an eight-channel head coil. Sequence parameters included three *B* = 0 volumes and 35 noncollinear diffusion encoded volumes yielding 2 mm isotropic resolution. Study staff preprocessed the resulting images using the FMRIB Software Library (FSL), v5.0.9, and the FSL AutoPtx plugin to compute tract-specific scalar metrics of white matter microstructure, including FA, MD, axial diffusivity (AD), and radial diffusivity (RD) for 27 white matter tracts. These included three brainstem tracts (middle cerebellar peduncle; left and right medial lemniscus), ten projection fibers (left and right corticospinal tracts and acoustic radiations, and bilateral anterior, posterior, and superior thalamic radiations), eight association fibers (bilateral superior and inferior longitudinal fasciculi, and bilateral inferior fronto-occipital and uncinate fasciculi), four limbic system fibers (left and right cingulate gyrus part of the cingulum and parahippocampal part of the cingulum), and two callosal fibers (forceps major and forceps minor) (de Groot et al., 2013). Online Supplementary Section 2 provides additional scan processing details. Researchers inspected all raw images, selected tractography data, and slice signal intensities to assess scan quality. Scans deemed poor quality (*n* = 1282) were excluded from analysis.

Following prior research on white matter microstructure, we focused our primary analyses on two measures, MD and FA (Lebel & Beaulieu, 2011; Smith et al., 2006). MD is a measure of the extent to which water molecules in white matter tissue move freely in all directions. FA assesses the extent to which white matter microstructure constrains water molecule diffusion in a single direction. In post hoc analyses, we also assessed AD and RD, which quantify how much water molecules are able to move in certain directions (Beaulieu, 2002). All four measures provide complementary information from which inferences about white matter microstructural anatomy can be made. As children age, MD values decrease, and FA values increase. Lower MD and higher FA values suggest more organized white matter, which in turn may enable more efficient neural functioning (Lebel & Deoni, 2018).

Because complex human behavior manifests from coordinated neural activity across several brain regions connected by several white matter tracts, we constructed ‘global’ measures of white matter microstructure via two different methods that incorporated information from all 27 tracts delineated by AutoPtx. We calculated the first measure by averaging and standardizing all tract-specific MD, FA, AD, and RD values (hereafter referred to as ‘unweighted’ mean global values) without regard to tract size. While tracts vary substantially in size, calculating arithmetic means in this way ensured each tract contributed equal information to our ‘global’ outcomes regardless of the tract's size, which enabled us to test our hypothesis that family functioning affects all (or nearly all) *white matter tracts* in the brain. Separately, we calculated standardized global FA, MD, AD, and RD values that explicitly account for tract size (hereafter referred to as ‘weighted’ mean global values) by weighting each tract's contribution to the overall global scalar by its volume. Because these weighted measures account for tract size, they enable us to test a somewhat different (though closely related) hypothesis that family functioning affects all (or nearly all) *white matter*, as opposed to white matter tracts. These alternative measures provide complementary information.

Separately, for the 24 tracts with analogs in both hemispheres (e.g. left and right uncinate fasciculus), we averaged and standardized measures from both hemispheres. For example, we averaged left and right MD values for each participant's uncinate fasciculi, resulting in a single mean MD value for the uncinate fasciculus. Because three tracts (middle cerebellar peduncle, forceps major, and forceps minor) do not have independent analogs in both hemispheres, this process resulted in 15 sets of tract-specific values used in our analyses.

#### Child behavior problems

In post-hoc analyses to explore the brain-behavior relationship, we used a measure of child behavioral problems. To assess behavior problems, mothers completed via postal questionnaire the Achenbach Child Behavior Checklist (CBCL/6-18) when participants were mean age 9.7 years (range 8.6–12.4). The CBCL asks how often children engage in 119 problematic behaviors on a three-point frequency scale (Achenbach & Rescorla, 2001). Following prior work, we summed responses to create continuous scores for total behavior problems (119 items, possible range 0–238) (Achenbach & Rescorla, 2001).

#### Covariates

Researchers retrieved sex from birth records along with birthdates, which we used to calculate age at MRI scan. Parents self-reported their national origin and ethnicity according to official definitions used by Statistics Netherlands, which we used to define and categorize child ‘ethnicity’ as European (non-Turkish), Turkish, Moroccan, Surinamese, and Other Ethnicity/National Origin. See online Supplementary Section 11 for more detail. Parents also self-reported their household income during pregnancy (< or ⩾ €2200/month); highest completed parental education level (less than high school equivalent; high school or intermediate vocational training; post-secondary or higher); parental history of psychosis (yes/no for each parent); maternal and paternal age at childbirth; maternal smoking during pregnancy (never; until pregnancy known; throughout pregnancy); and parental psychopathology symptoms (continuous sum scores for each parent) at two timepoints: (1) prenatally (for models of prenatal family functioning; measured using the full 53-item Brief Symptom Inventory (BSI)); and (2) at child age 3 years (for models of mid-childhood family functioning; measured using a subset of 21 BSI items available at that timepoint) (Derogatis & Melisaratos, 1983).

### Statistical analyses

We assessed and removed as appropriate outliers in MD, FA, AD, and RD using standard methods (*n* = 82 removed). Online Supplementary Section 3 details our methods and rationale.

To investigate whether family functioning was associated with our primary measures of white matter microstructure (i.e. both unweighted and weighted global MD and FA), we used ordinary least squares-estimated linear regression. We imposed a hierarchical structure to these analyses with primary models examining global outcomes and subsequent models evaluating specific tracts. For each outcome, we fit (1) minimally adjusted models accounting for each child's age at scan, sex, and ethnicity/national origin; and (2) fully adjusted models adding all other covariates listed above. We ran separate models to assess associations with prenatal and mid-childhood family functioning, after which we considered models including functioning scores from both timepoints simultaneously. We weighted all models to account for differential attrition by sociodemographic characteristics using inverse probability weights described below. In post hoc analyses, we followed the same analysis plan for both unweighted and weighted global AD and global RD.

We conducted several sensitivity analyses with respect to our primary outcomes (i.e. unweighted and weighted global MD and FA). First, we evaluated whether prenatal family functioning modified effects of mid-childhood family functioning by incorporating an interaction term between prenatal and mid-childhood functioning scores using continuous measures in fully adjusted models. Second, we evaluated associations between global outcomes and mean family functioning by averaging functioning scores from both prenatal and mid-childhood timepoints. Third, because there was substantial left skew in the functioning score distributions (see below for more detail), we fit fully adjusted piecewise continuous linear spline models of prenatal functioning and our global outcomes. Based on *a priori* considerations of the family functioning scale and score distributions in our sample, we initially modeled a knot at a score of 3.0, after which we iteratively modeled alternative knots below 3.0 in score decrements of 0.1. Finally, in post-hoc exploratory analyses, we tested associations of (1) prenatal family functioning with child behavior problems (CBCL total behavior problem score) and (2) global FA and MD with child behavior problems in fully adjusted models using the same modeling strategy outlined above.

After modeling our data, we interpreted the primary model results consistent with guidance provided by the American Statistical Association based on effect magnitudes, effect directions, and 95% confidence intervals in lieu of binary indicators of statistical significance, though we provide *p* values as an interpretive heuristic (Wasserstein & Lazar, 2016). For primary models, which test only two outcomes (i.e. global MD and global FA), we do not adjust these *p* values for multiple comparisons. However, for secondary, tract-specific results, we augment effect magnitudes and confidence intervals with statistical significance indicators after adjusting *p* values for multiple tests via the Simes/Benjamini-Hochberg procedure, a method that controls the false discovery rate when assuming non-negative correlation among estimates (15 tracts, 15 comparisons) (Benjamini & Hochberg, 1995; Simes, 1986).

### Missing data

To account for differential attrition by sociodemographic characteristics, we calculated the inverse probability of attrition weights (IPWs). We deemed lost to follow-up any participant enrolled at baseline but excluded from our analytic sample for any reason. Separately, we multiply imputed missing exposure and covariate data using chained equations to construct 50 imputed datasets, then combined imputation-specific mean and variance measures using Rubin's Rules (Rubin, 1996). Online Supplementary Sections 4 and 5 detail our IPW and imputation models.

## Results

### Analytic sample characteristics

Included *v.* excluded participants were more likely to be of European ethnicity/national origin (71% *v.* 58%); to have parents with at least advanced vocational training or a bachelor's degree (63% *v.* 44%); to be from higher-income households (52% *v.* 32%); and to be born to older mothers (mean maternal age at birth 31.7 years *v.* 29.8 years). Nevertheless, our final analytic sample was socioeconomically diverse. Nearly half (48%) of participants were from households earning less than €2200/month at study enrollment (approximately $33 000/year at exchange rates in 2004), while over one-third of participants were born to parents with no more than a high school equivalent education.

Table 1 details sociodemographic characteristics in our analytic sample according to family functioning scores. Mothers of European children reported higher family functioning at both timepoints than mothers of children of other ethnicities, as did mothers of higher-income and education households. Prenatal and mid-childhood scores were moderately correlated, *r* = 0.38. Functioning scores at both timepoints were left skewed. Prenatal mean and median scores were 3.49 (s.d. = 0.46) and 3.58, respectively, with 81% of mothers in the analysis sample reporting scores greater than 3.0 (scale range 1.0–4.0). Similarly, mid-childhood mean and median scores were 3.50 (s.d. = 0.42) and 3.58, respectively, with 83% of mothers reporting mid-childhood scores above 3.0. These score distributions in our analytic sample were consistent with those found in prior studies, which report that families from community-based samples generally report high levels of functioning and low levels of conflict (Epstein et al., 1983; Mansfield, Keitner, & Dealy, 2015; Miller, Epstein, Bishop, & Keitner, 1985). See online Supplementary Section 6 for mean outcome measures by sociodemographic characteristics.

**Table 1.** Distribution of exposure measures by participant characteristics in the final analytic sample. *n* = 2727^a^

		Family functioning scores^b^					
		Prenatal			Mid-childhood		
	%	*x̅*	*s*	*p^c^*	*x̅*	*s*	*p^c^*
Total sample	100	3.49	0.46		3.50	0.42	
Sex				0.36			0.09
Female	51	3.49	0.46		3.52	0.41	
Male	49	3.48	0.45		3.49	0.43	
Ethnicity/National origin				< 0.01			< 0.01
Dutch/Other European	71	3.56	0.42		3.56	0.40	
Turkish	5	3.26	0.47		3.28	0.50	
Moroccan	4	3.26	0.47		3.28	0.40	
Surinamese	7	3.27	0.50		3.40	0.45	
Other	13	3.31	0.52		3.41	0.43	
Highest household education				< 0.01			0.01
Less than high school equivalent	4	3.17	0.48		3.22	0.47	
High school or intermediate vocational training	33	3.37	0.49		3.45	0.43	
Adv. vocational training, bachelor's, or higher	63	3.57	0.41		3.56	0.40	
Household income				< 0.01			< 0.01
€2200/month or less	48	3.35	0.49		3.42	0.45	
More than €2200/month	52	3.59	0.40		3.58	0.38	

^a^This table is based on observed values for each characteristic and does not account for missing data.

^b^Family functioning scores are based on the McMaster Family Assessment Device – General Functioning Subscale, and they range from 1 to 4.

^c^*p* values for differences in mean family functioning scores by sociodemographic category are from *t* tests or χ^2^ tests, as appropriate.

### Global outcomes

In fully adjusted models, prenatal family functioning was positively associated with preadolescent global FA (both unweighted and weighted) and negatively associated with global MD (unweighted, with more modest evidence with respect to weighted global MD). See Table 2. For example, *β*_unweighted global FA_ = 0.11 (95% CI 0.00, 0.21) and *β*_unweighted global MD_ = −0.15 (95% CI −0.28, −0.03). For context, these estimates can be compared to those of other known contributors to white matter microstructural differences. The estimated magnitudes listed above for the effects of a one-unit increase in prenatal functioning score for unweighted global outcomes were approximately 52 and 82% of those associated with a one-year increase in scan age in models for global FA and MD, respectively. See online Supplementary Section 7 for beta estimates from all other covariates in these models. In contrast, we found no evidence of an association between mid-childhood functioning and either global measure of white matter microstructure. Notably, in models of mid-childhood functioning adjusting for prenatal functioning, prenatal functioning effect estimates were broadly consistent with those from models that did not include functioning scores from both time points. For example, in weighted, fully adjusted models simultaneously including both mid-childhood and prenatal functioning scores, effect estimates for prenatal functioning were *β*_global FA_ = 0.14 (95% CI 0.02, 0.25) and *β*_global MD_ = −0.11 (95% CI −0.23, 0.02). Online Supplementary Section 8 includes post hoc model results for global RD and AD, which suggest global RD – but not AD – is associated with prenatal family functioning.

**Table 2.** Associations between family functioning and two mean measures (unweighted and weighted by tract volume) of global fractional anisotropy and global mean diffusivity in preadolescence.^d^ *n* = 2727

		Global FA			Global MD		
		*β*	95% CI	*p*	*β*	95% CI	*p*
Prenatal							
Min. adjusted^b^	Unweighted^a^	0.14	(0.04, 0.24)	0.007	−0.12	(−0.24, 0.00)	0.005
Min. adjusted	Weighted^a^	0.13	(0.03, 0.24)	0.014	−0.09	(−0.20, 0.03)	0.140
Fully adjusted^c^	Unweighted	0.11	(0.00, 0.21)	0.049	−0.15	(−0.28, −0.03)	0.014
Fully adjusted	Weighted	0.12	(0.01, 0.23)	0.040	−0.10	(−0.23, 0.02)	0.091
Mid-childhood, not adjusted for prenatal score							
Min. adjusted	Unweighted	−0.01	(−0.13, 0.10)	0.810	−0.01	(−0.13, 0.11)	0.908
Min. adjusted	Weighted	−0.02	(−0.14, 0.09)	0.709	−0.01	(−0.12, 0.11)	0.894
Fully adjusted	Unweighted	−0.04	(−0.16, 0.08)	0.500	−0.02	(−0.14, 0.10)	0.715
Fully adjusted	Weighted	−0.04	(−0.16, 0.08)	0.515	−0.01	(−0.13, 0.11)	0.826
Mid-childhood, adjusted for prenatal score^e^							
Min. adjusted	Unweighted	−0.07	(−0.19, 0.06)	0.282	0.04	(−0.09, 0.16)	0.556
Min. adjusted	Weighted	−0.07	(−0.20, 0.05)	0.235	0.02	(−0.10, 0.14)	0.694
Fully adjusted	Unweighted	−0.07	(−0.20, 0.05)	0.247	0.02	(−0.10, 0.14)	0.748
Fully adjusted	Weighted	−0.08	(−0.20, 0.05)	0.235	0.02	(−0.11, 0.14)	0.809

^a^‘Unweighted’ global measures weight each tract equally, i.e. they are the standardized arithmetic mean FA and MD values of all 27 tracts delineated by AutoPtx regardless of size. ‘Weighted’ measures are weighted by tract volume.

^b^Minimally adjusted models include covariates for child age at scan, sex, and ethnicity/national origin.

^c^Fully adjusted models account for child age at scan, sex, ethnicity/national origin, household income, highest level of parental education, maternal and paternal history of psychosis, prenatal maternal and paternal psychopathology symptoms (for prenatal models), early-childhood maternal and paternal psychopathology symptoms (for mid-childhood models), maternal and paternal age at child's birth, and child in utero exposure to smoking.

^d^All models use inverse probability of attrition weights to account for differential attrition from baseline.

^e^Results for possible associations between mid-childhood functioning and the global outcomes that appear under the subheading ‘Mid-childhood, adjusted for prenatal score’ are from models that simultaneously include both mid-childhood and prenatal functioning scores.

### Tract-specific outcomes

**Fig. 1.**
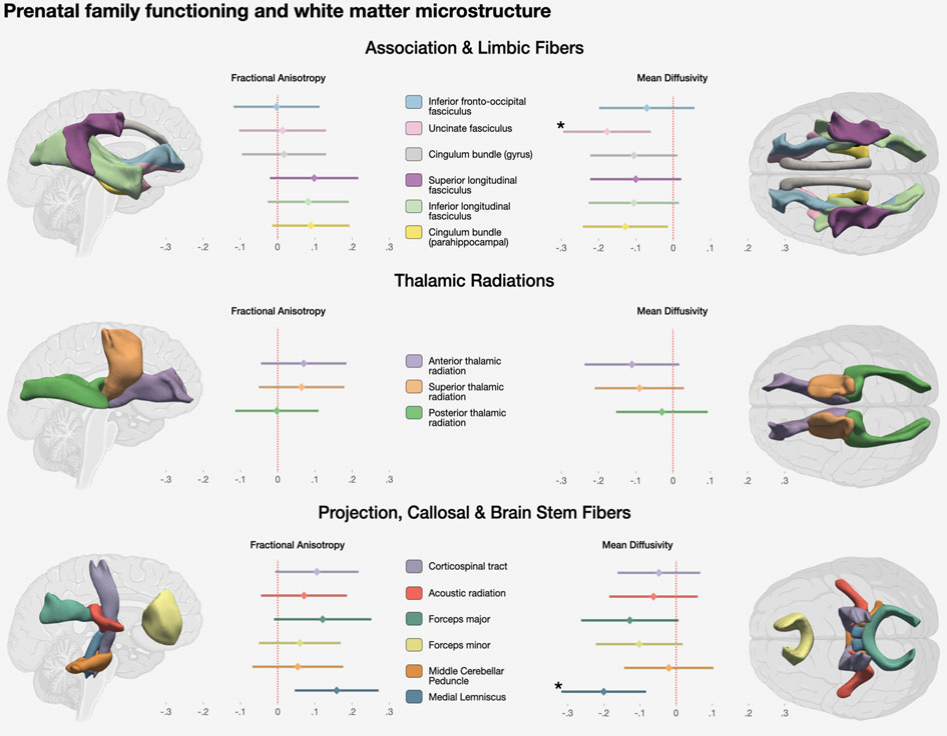
Associations between prenatal family functioning and tract-specific FA and MD. Estimates are from fully adjusted models accounting for child age at MRI scan, sex, ethnicity/country of origin, household income, highest level of parental education achieved, maternal and paternal history of psychosis, prenatal maternal and paternal psychopathology symptoms, maternal and paternal age at child's birth, and child in utero exposure to smoking. All models use inverse probability of attrition weights. Coefficient plot estimates are standard deviation differences associated with a one-point increase in prenatal family functioning score (score range 1–4). *Starred estimates remain statistically significant after FDR adjustment.

Exploratory tract-specific models revealed negative associations between prenatal functioning and MD in the uncinate fasciculus, medial lemniscus, and cingulum bundle (parahippocampal part); however, only the first two associations (uncinate fasciculus and medial lemniscus) survived adjustment for multiple testing (Table 3, Fig. 1). The remaining tract-specific MD effect estimates had larger standard errors and thus did not evince associations based on statistical significance, but all 15 MD effect estimates were uniform in direction (Fig. 1). A similar pattern emerged from models assessing prenatal functioning and tract-specific FA. Effect estimates were nearly uniform in the direction of a positive relationship with prenatal family functioning, though none were statistically significant after adjustment for multiple testing, including the estimate for medial lemniscus FA (*β*_ML FA_ = 0.16; 95% CI 0.05, 0.27; uncorrected *p* value = 0.006).

**Table 3.** Associations between prenatal family functioning and tract-specific measures of white matter microstructure.^a,b^ *n* = 2727

	Fractional anisotropy^c^			Mean diffusivity^c^		
	*β*	95% CI	*p*	*β*	95% CI	*p*
Association fibers						
Superior longitudinal fasciculus	0.10	(−0.02, 0.22)	0.105	−0.10	(−0.22, 0.02)	0.107
Inferior longitudinal fasciculus	0.08	(−0.03, 0.19)	0.138	−0.11	(−0.23, 0.02)	0.087
Inferior fronto-occipital fasciculus	−0.00	(−0.12, 0.11)	0.962	−0.07	(−0.20, 0.06)	0.278
Uncinate fasciculus	0.01	(−0.10, 0.13)	0.822	−0.18	(−0.30, −0.06)	0.003*
Limbic system fibers						
Cingulum (cingulate gyrus part)	0.02	(−0.10, 0.13)	0.764	−0.11	(−0.22, 0.01)	0.078
Cingulum (parahippocampal part)	0.09	(−0.01, 0.19)	0.091	−0.13	(−0.24, −0.01)	0.027
Projection fibers						
Corticospinal tract	0.11	(−0.01, 0.22)	0.066	−0.05	(−0.16, 0.07)	0.411
Acoustic radiation	0.07	(−0.05, 0.19)	0.232	−0.06	(−0.19, 0.06)	0.315
Anterior thalamic radiation	0.07	(−0.04, 0.18)	0.233	−0.11	(−0.24, 0.02)	0.090
Superior thalamic radiation	0.06	(−0.05, 0.18)	0.275	−0.09	(−0.21, 0.03)	0.140
Posterior thalamic radiation	−0.00	(−0.11, 0.11)	0.968	−0.03	(−0.15, 0.09)	0.630
Callosal fibers						
Forceps major	0.12	(−0.01, 0.25)	0.071	−0.13	(−0.26, 0.01)	0.062
Forceps minor	0.06	(−0.05, 0.17)	0.289	−0.10	(−0.22, 0.02)	0.095
Brainstem tracts						
Middle cerebellar peduncle	0.05	(−0.07, 0.18)	0.386	−0.02	(−0.14, 0.10)	0.745
Medial lemniscus	0.16	(0.05, 0.27)	0.006	−0.20	(−0.32, −0.08)	0.001*

^a^Fully adjusted models account for child age at scan, sex, ethnicity/national origin, household income, highest level of parental education achieved, maternal and partner history of psychosis, maternal and partner psychopathology symptoms, maternal and paternal age at child's birth, and child in utero exposure to smoking.

^b^All models are weighted to account for differential attrition from baseline by sociodemographic characteristics.

^c^MD and FA values are averaged across hemispheres where appropriate and standardized.

*Starred results remain statistically significant after adjustment for multiple comparisons.

### Sensitivity analyses

In fully adjusted models of both unweighted and weighted global MD and FA, we found no evidence of statistical interaction between prenatal and mid-childhood functioning scores. Interaction terms were *β*_unweighted global FA_ = 0.11 (95% CI −0.15, 0.37) and *β*_unweighted global MD_ = −0.00 (95% CI −0.30, 0.29). Separately, fully adjusted models of mean family functioning scores yielded only marginal or no evidence that mean functioning was associated with either outcome: *β*_unweighted global FA_ = 0.05 (95% CI −0.08, 0.20) and *β*_unweighted global MD_ = −0.15 (95% CI −0.30, 0.01). Piecewise continuous linear spline models suggested effects of greater magnitudes for lower prenatal functioning scores, i.e. scores between 1.0 and 3.0. See online Supplementary Section 9 for estimates from these models. However, given the relatively fewer number of participants with lower functioning scores, these effect estimates were uncertain. Among the relatively greater number of participants with higher functioning scores (i.e. above 3.0), effect estimates were smaller.

### Exploratory behavioral analyses

In fully adjusted models, higher prenatal functioning was associated with lower preadolescent CBCL total problem scores: *β* = −3.37 (95% CI −5.48, −1.26) for each one-unit increase in prenatal family functioning score. Separately, we found no statistical evidence of associations between global measures of white matter microstructure and CBCL total behavior problem scores in this sample. Online Supplementary Section 12.

## Discussion

This study provides evidence that early-life family functioning may affect white matter neurodevelopment. Specifically, more positive prenatal family environments (i.e. supportive and accepting families with high problem-solving capacity) were associated with higher FA and lower MD, on average, across the brain's white matter in preadolescence. While the effect estimate magnitudes were relatively small in absolute terms for all global outcomes, they can be compared to other known contributors to white matter microstructure. For example, the difference in unweighted global MD associated with a one-unit increase in prenatal family functioning score was about four-fifths of the difference associated with a one-year increase in scan age. The three-unit range of the family functioning scale between low *v.* high functioning families (i.e. from 1 to 4) renders these estimates more substantial, and prior research suggests similar differences in white matter microstructure can manifest clinically as child behavior problems (Lebel & Deoni, 2018; Waller et al., 2017).

In contrast to our prenatal findings, we found no evidence suggesting a relationship between mid-childhood family functioning and our global outcomes. One possible explanation for these diverging results relates to the decreasing relative importance of the family environment to children's broader social environment over time. When children are very young, their social environment consists almost exclusively of their family. However, as they grow older, they attend school, spend more time with friends, and establish influential relationships outside the family. In this way, the family environment, while still exceedingly important to healthy child development, may be somewhat less impactful over time as the child's social environment diversifies. Avants et al. (2015) propose a similar rationale to explain results of their study, in which measures of a stimulating home environment assessed at age 4 – but not measures assessed at age 8 – were associated with cortical thickness in late adolescence (Avants et al., 2015).

Secondary analyses support the hypothesis that effects of prenatal family functioning on white matter microstructure may be widespread throughout the brain. For example, with respect to MD, only associations with the uncinate fasciculus and medial lemniscus remained statistically significant after adjustment for multiple testing, but the uniform direction of the remaining tracts' estimates suggests a model of global rather than tract-specific effects. Moreover, if effects were tract-specific (rather than global), one might postulate that the uncinate fasciculus and medial lemniscus share a common functional role, but they do not. The uncinate fasciculus connects the brain's temporal and frontal lobes and is involved in memory, language, and social-emotional processing, while the medial lemniscus is a brainstem tract involved in sensory information transport (Purves et al., 2018); Von Der Heide, Skipper, Klobusicky, & Olson, 2013). Thus, our tract-specific analyses suggest prenatal family functioning may have global effects.

Our findings are consistent with the limited prior work in this area. In the only other study assessing prenatal experiences and white matter microstructure in a population-based cohort, Jensen et al. (2018) reported increased maternal prenatal stressful experiences were associated with decreased splenium magnetization transfer ratio (MTR), a marker of lower white matter microstructure, in early adulthood (Jensen et al., 2018). Our findings correspond with Jensen et al. (2018) because they reported *stressful* prenatal experiences were associated with *less* microstructure, while our study reports *enriched* prenatal environments are associated with *more* microstructure.

Moreover, our results support prior investigations reporting that positive parenting practices and relationships confer neurodevelopmental advantages associated with decreased risky behavior. Specifically, in our study, higher levels of prenatal family functioning were associated with both greater white matter microstructure and – in post-hoc analyses – lower overall behavior problems. While white matter microstructure was not associated with behavior problems in our post-hoc analyses, this brain-behavior relationship has been established previously by several studies that were designed specifically to identify brain phenotypes of behavior problems (Bolhuis et al., 2019; Lebel & Deoni, 2018; Waller et al., 2017). Our study, in contrast, was designed to assess relationships between early-life family functioning and brain structure, and as such, it builds on prior studies investigating how the family environment may impact neurodevelopment.

Notably, because many of these prior studies assess the family environment after the children are born, they are vulnerable to reverse causation, since child behavior likely influences family functioning. Our study, however, found similar effects using a family functioning measure obtained before the child's birth, thereby reducing concerns about recall bias and reverse causation. Together, these findings warrant additional investigation exploring whether and to what extent positive prenatal and early-life experiences induce lasting white matter microstructural changes beyond those associated with the mere absence of negative early-life experiences. Findings from such studies would be important because they may have implications for child development policy and public mental health. Gaining a greater understanding of the neural mechanisms mediating relationships between specific facets of the early-life social environment and child mental wellbeing can clarify how the brain changes in response to specific types of experiences, including experiences positive in utero, which are not commonly considered within the context of child mental health. Our study contributes to a broader body of research that may help provide insight into the types and timing of interventions that enable children to maximize their potential.

Our prenatal measure of the family environment is unlikely to measure the prenatal environment exclusively. More likely, it captures the perinatal and early-childhood family environment, spanning some period both before and after the child's birth. Interestingly, we found prenatal and mid-childhood functioning scores were only moderately correlated (*r* = 0.38), suggesting that the family environment may change modestly through the child's first six years. Follow-up research may investigate to what extent family functioning fluctuates during this time period.

Jensen et al. (2018) propose at least three complementary mechanisms that could explain how prenatal stress may affect white matter microstructure. The first is the balance between neurogenesis – which mostly ends just after birth (if not before) and does not meaningfully extend into mid-childhood – and apoptosis. Studies in humans and other animals suggest both processes are experience-dependent and affect neuronal density. Stressful environments – and the stress hormones they produce – may reduce neuronal density by decreasing neurogenesis and increasing apoptosis, while enriched environments may do the opposite (Jensen et al., 2018; Lebel & Deoni, 2018). Increased neuronal density could result in higher FA and lower MD (Jensen et al., 2018). Importantly, if the associations observed in this study (1) reflect biological reality and (2) are caused by a change in neurogenesis and apoptosis, then neuroplastic processes might be able to compensate partially for these prenatal effects later in a child's life, but they would be unable to undo them entirely because neurogenesis largely ends prior to or just after birth.

Another possible mechanism is altered developmental myelination, or the process by which axons develop an insulating myelin sheath to enhance their efficiency. Enriched environments have been associated with increased FA and decreased MD, which suggest greater myelination (Jensen et al., 2018). Healthier family functioning may have effects similar to those of enriched environments. A third potential mechanism relates to changes in axonal diameter and the thickness of the myelin sheath. Larger axons have thinner myelin sheaths compared to smaller axons. Because enriched environments entail novel and healthy stimuli, they may increase neuronal activity and promote axonal growth (Jensen et al., 2018). Both FA and MD may be influenced by these changes, such that a greater density of large-diameter axons (perhaps resulting from enriched environments) would manifest as higher FA and lower MD.

Our study has limitations. First, with only one DWI scan per participant, we cannot assess changes in neurodevelopmental trajectories due to our exposures. Studies with repeated imaging measures over time would enable more direct assessment of brain changes throughout childhood (Bhanot et al., 2021). Relatedly, it is possible that associations with positive prenatal functioning may instead be due to healthy family environments that remain somewhat constant into preadolescence, though our null findings with respect to mid-childhood functioning suggest this possibility may be less likely. Second, our sample included few families reporting low functioning scores. This inhibits our ability to examine effects of scores at the low end of the continuum. Third, confounding may have biased our results if, for example, certain parental genetic profiles influence parents' assessment of their family's functioning while also affecting their child's white matter structure. Similarly, reverse causation may occur if a young infant's brain structure and resulting behavior influence mid-childhood family functioning. We partly addressed this concern by adjusting for maternal and paternal psychopathology symptoms and psychosis history. Fourth, differential attrition in the cohort by sociodemographic characteristics may have induced selection bias. We also excluded several participants due to poor scan quality, which can be patterned by child behavior and sociodemographic profiles. These two challenges are inherent in nearly all large pediatric neuroimaging studies, and as such, we implemented inverse probability of attrition weights to reduce these threats of bias. Selection in utero may also induce survival bias, wherein frail fetuses of mothers reporting high prenatal family functioning may have survived and been included in our analyses when they would not have done so if they were from lower-functioning families (Nobles & Hamoudi, 2019). In turn, prenatal functioning effect magnitudes are likely to be underestimated. Finally, our analyses do not account for possible partial volume effects related to head size that may influence DTI scalar metrics (Vos, Jones, Viergever, & Leemans, 2011).

Separately, we note two limitations with respect to the interpretation of these findings. First, the applicability of these results to target populations in other countries (i.e. the transportability of these results) may be affected by differences in various facets of each country's social environment. For example, the social construction of ‘race’ and/or ‘ethnicity’ in other countries may differ from the Dutch construction of ‘ethnicity’, and these differences should be considered when assessing the transportability of the results. Second, as population neuroimaging cohorts grow larger, studies may be able to identify somewhat smaller effect magnitudes than were possible in smaller studies. In turn, previously used heuristics based on effect sizes (e.g. Cohen's d, etc.) may not be well-suited to studies of this type (Owens et al., 2021). We have partially addressed this challenge by comparing the effect estimate magnitudes to other known contributors (e.g. age) to white matter microstructure.

Our study also has several strengths. First, we used a longitudinal design, leveraging prospectively collected exposure data predating the child's birth and outcomes measured fully ten years later, which enabled us to investigate relatively long-term effects of the perinatal family environment. We also avoid many challenges associated with studies using maternal reports of both exposures and outcomes (e.g. behavior measures) by using objective outcomes calculated from DWI scans. Finally, this study is nested within a large, population-based birth cohort, which reduces the risk of selection bias common to many neuroimaging studies relying on case-control designs.

## Conclusions

In a socioeconomically diverse sample of 2727 children, higher prenatal family functioning – a measure of the perinatal family environment – was associated with greater white matter microstructure approximately ten years later, suggesting healthy perinatal family functioning may confer lasting neurodevelopmental advantages. Our results also suggest the emphasis on parenting practices in family-focused child neurodevelopmental research may be too narrow, and that more general measures of family functioning may capture important health-relevant dimensions of the family environment. Subsequent neurodevelopmental studies of family functioning should consider developing tools to better assess variation at the lower and higher ends of the spectrum, and they should emphasize the participation of low-functioning families. Capturing positive aspects of early-life family functioning may provide important insight into novel pathways by which facets of the social environment become biologically embedded and link to child well-being.

## Supplementary material

For supplementary material accompanying this paper visit https://doi.org/10.1017/S0033291722001404. click here to view supplementary material
