# Supplementary material for: Long-term associations between early-life family functioning and preadolescent white matter microstructure
Source: Psychol Med. 2022 May 25;53(10):4528–38. doi: 10.1017/S0033291722001404 (PMC10388303; doi:10.1017/S0033291722001404)
Supplement: Supplementary file 1 [file S0033291722001404sup001.docx]

**Appendix of Supplemental Information**

Section 1.


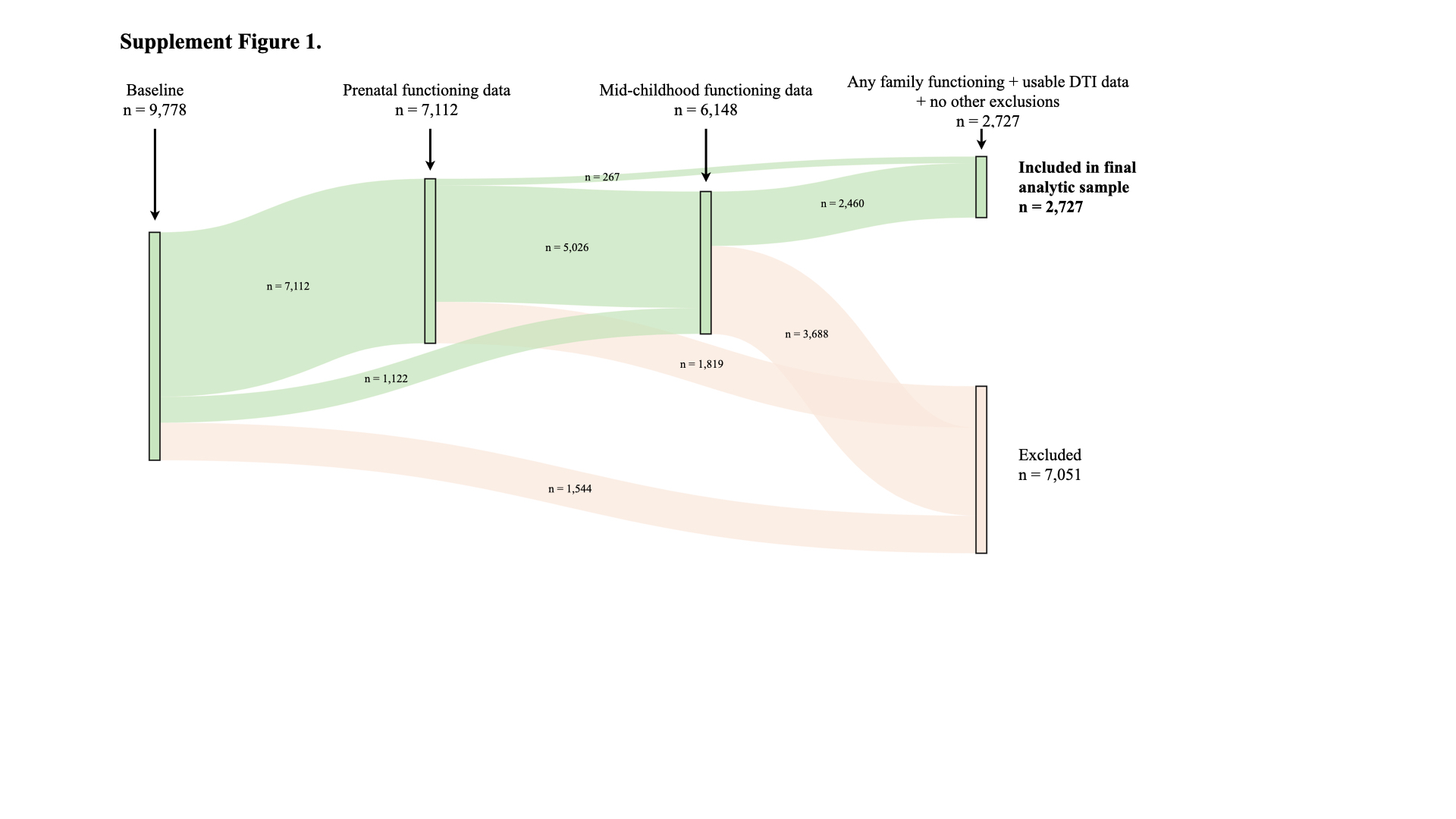


| **Supplement Table 1. Reasons for excluding participants from analytic sample.** | |
| --- | --- |
| 1,544 | Missing both prenatal and mid-childhood family functioning data |
| 4,026 | No MRI / DTI scan data |
| 1,282 | Unusable DTI scan data (poor quality image) |
| 12 | Incidental finding on MRI scan |
| 8 | In utero exposure to heroin or cocaine |
| 37 | Randomly selected twin removed |
| 5 | Extremely low birthweight, < 1,000 g |
| 55 | Missing DTI scalar data for at least one tract |
| 82 | Outlier DTI scalar values |
| **7,051** | **Total excluded participants** |
|  |  |
| *Note: “MRI” = magnetic resonance imaging; “DTI” = diffusion tensor imaging, a specific type of MRI.* | |

Section 2. Brain imaging details.

All MRI and DTI brain scans were acquired by a GE MR-750W scanner (General Electric Healthcare, Chicago, IL) at 3T with an eight-channel head coil.(White et al., 2018) Sequence parameters included 2 mm isotropic resolution and 35 diffusion-weighted volumes. Study staff preprocessed the resulting images using the FMRIB Software Library (FSL), v5.0.9, which stripped non-brain tissue, corrected for artifacts from eddy currents and head motion, and fit a diffusion tensor to each voxel using the RESTORE method from the Camino diffusion MRI toolkit. This pipeline calculated fractional anisotropy (FA), mean diffusivity (MD), axial diffusivity (AD), and radial diffusivity (RD) metrics for each voxel.

Next, study researchers conducted fully automated probabilistic fiber tractography on each participant’s diffusion-weighted image in native space using the AutoPtx plugin for FSL(de Groot et al., 2013). This method generates subject-specific, probabilistic representations of 27 large white matter tracts that can be consistently and robustly identified across brain regions. The process identifies each tract’s connectivity distribution, normalizes it given the number of successful seed-to-target attempts, and then removes voxels unlikely to be part of the tract’s distribution. Thereafter, the process automatically computes tract-specific scalar metrics (MD, FA, AD, RD) of microstructural properties by weighting voxel-specific metrics by the probability that each voxel is part of the specific tract. To ensure the quality of all scans and reconstructions, researchers visually inspected all raw images and examined signal intensity in each slice to assess attenuation by various artifacts. They also visually inspected all probabilistic tractography data. Scans deemed poor at any point in the quality control process were excluded from analysis.

Section 3. Outlier analyses.

We assessed statistical outliers in four measures of white matter microstructure: tract-specific MD, FA, AD, and RD. Though our primary outcomes are composite MD and FA metrics, we included AD and RD outcomes in the outlier analyses because they are based more directly on tensor eigenvalues describing diffusion anisotropy than MD and FA and therefore may be less likely to obscure extreme values. For example, while MD is the mean of all three tensor eigenvalues (λ_1_, λ_2_, and λ_3_), RD is merely the mean of two (λ_2_ and λ_3_), and AD is simply λ_1_, where λ_1_ > λ_2_ > λ_3_. We excluded participants with any tract-specific MD, FA, AD, or RD value greater than 5 standard deviations from the mean value for each respective tract because such values are either (1) biologically implausible or (2) so far from the sample means that they likely represent significant pathology or brain structural abnormality.

Section 4. Inverse probability of attrition weights.

We defined participants “lost to follow up” as those enrolled at baseline (either prior to or at birth) but excluded from our analysis sample for any reason. Thus, we deemed participants lost to follow up if they chose not to participate in later rounds of data collection or if they did participate but their MRI scan data was unusable. To calculate our IPWs, we identified a broad set of variables theorized to predict who among originally enrolled participants satisfied our inclusion criteria. We used multiple imputation by chained equations (predictive mean matching for all variables, knn = 10, burn-in = 25) to address missing data in these variables, resulting in 50 imputed datasets. Thereafter, we fit a logistic regression model using these variables to predict the likelihood of each enrolled participant’s inclusion in our analysis sample. The predictive accuracy of this model yielded an area under the receiver operating characteristic curve (AUC of ROC curve) of 0.800 (SE = 0.005). Last, we calculated IPWs for use in later analyses. Unstabilized weights had a mean of 3.22 and ranged from 1.36 to 17.15.

Section 5. Multiple imputation models.

We imputed missing exposure and covariate data. The proportion of missing data for most covariates was low to moderate (e.g., 12% for paternal age at birth), with the exception of household income, for which we were missing 20% of data. We used the ‘mi impute chained’ package in Stata 16.0/MP. For continuous variables, we specified linear regression models. For ordinal and categorical variables, we specified predictive mean matching models, knn = 10 (i.e., 10 donor values). We specified a 25-iteration burn-in period for each chain to ensure convergence to a stationary distribution. Models included all outcomes as right-hand side variables with no missing data. We imputed 50 imputed datasets and combined the resulting estimates using Rubin’s Rules (Rubin, 1996).

Section 6. Mean FA and MD values by sociodemographic characteristics.

On average, girls had lower unweighted global MD and FA scores than boys (*p*-values *<* 0.001 for both outcomes). European children had higher global FA values than children of other countries of origin (*p* < 0.001). Children of more socially advantaged households had higher global FA values than their less advantaged counterparts (*p* = 0.001 for both parental education and household income). No differences in global MD by country of origin, parental education, or household income were evident.

| **Supplement Section 6. Distribution of unweighted global FA and MD by participant characteristics in the final analytic sample. n = 2,727.** | | | | | | | | |
| --- | --- | --- | --- | --- | --- | --- | --- | --- |
|  |  |  |  |  |  |  |  |  |
|  |  | Global FA | |  |  | Global MD | |  |
|  | % | *x̅* | *s* | *p* |  | *x̅* | *s* | *p* |
|  |  |  |  |  |  |  |  |  |
| Total Sample | 100 | 0.00 | 1.00 |  |  | 0.00 | 1.00 |  |
| Sex |  |  |  | < 0.001 |  |  |  | < 0.001 |
| Female | 51 | -0.10 | 1.01 |  |  | -0.12 | 0.98 |  |
| Male | 49 | 0.11 | 0.98 |  |  | 0.12 | 1.01 |  |
| Ethnicity / Country of origin |  |  |  | < 0.001 |  |  |  | 0.278 |
| Dutch / Other European | 71 | 0.06 | 1.00 |  |  | -0.01 | 0.99 |  |
| Turkish | 5 | -0.20 | 0.98 |  |  | 0.02 | 1.03 |  |
| Moroccan | 4 | -0.14 | 0.90 |  |  | 0.20 | 0.95 |  |
| Surinamese | 7 | -0.24 | 1.01 |  |  | -0.02 | 1.01 |  |
| Other | 13 | -0.11 | 0.97 |  |  | 0.01 | 1.03 |  |
| Highest Household Education |  |  |  | 0.001 |  |  |  | 0.531 |
| Less than high school equivalent | 4 | -0.10 | 0.92 |  |  | -0.10 | 0.94 |  |
| High school / intermed. vocation training | 33 | -0.09 | 1.01 |  |  | -0.02 | 1.06 |  |
| Adv. vocation training, bachelor's, higher | 63 | 0.07 | 0.99 |  |  | 0.01 | 0.97 |  |
| Household Income |  |  |  |  |  |  |  | 0.934 |
| €2200 / month or less | 48 | -0.06 | 1.01 | 0.001 |  | 0.00 | 1.03 |  |
| More than €2200 / month | 52 | 0.06 | 0.98 |  |  | 0.00 | 0.97 |  |
|  |  |  |  |  |  |  |  |  |
| *a. This table is based on observed values for each characteristic and does not account for missing data.* | | | | | | | | |
| *b. p-values are from one-way ANOVA F-tests for differences in outcomes by each respective participant characteristic.* | | | | | | | | |
|  |  |  |  |  |  |  |  |  |

Section 7.

| **Supplement Section 7. Full model results from fully adjusted models assessing associations between prenatal family functioning, “unweighted” global fractional anisotropy (FA), and “unweighted” global mean diffusivity (MD) in preadolescence. n = 2,727.** | | | | | | | |
| --- | --- | --- | --- | --- | --- | --- | --- |
|  | Global FA | | |  | Global MD | | |
|  | β | 95% CI | *p* |  | β | 95% CI | *p* |
|  |  |  |  |  |  |  |  |
| Family functioning | 0.11 | (0.00, 0.21) | 0.05 |  | -0.15 | (-0.28, -0.03) | 0.01 |
|  |  |  |  |  |  |  |  |
| Age | 0.20 | (0.13, 0.27) | < 0.01 |  | -0.19 | (-0.26, -0.12) | < 0.01 |
| Sex | 0.20 | (0.11, 0.28) | < 0.01 |  | 0.28 | (0.19, 0.37) | < 0.01 |
| Ethnicity / Country of origin |  |  |  |  |  |  |  |
| Dutch / Other European | ref | | |  | ref | | |
| Turkish | -0.14 | (-0.33, 0.06) | 0.17 |  | 0.01 | (-0.19, 0.20) | 0.94 |
| Moroccan | -0.14 | (-0.33, 0.05) | 0.02 |  | 0.23 | (0.00, 0.46) | 0.05 |
| Surinamese | -0.27 | (-0.45, -0.08) | < 0.01 |  | -0.03 | (-0.21, 0.14) | 0.73 |
| Other | -0.11 | (-0.24, 0.02) | 0.10 |  | -0.03 | (-0.17, 0.11) | 0.63 |
| Highest Household Education |  |  |  |  |  |  |  |
| Less than high school equivalent | ref | | |  | ref | | |
| High school / intermed. vocation training | -0.14 | (-0.50, 0.22) | 0.45 |  | 0.19 | (-0.17, 0.55) | 0.30 |
| Adv. vocation training, bachelor's, higher | -0.13 | (-0.50. 0.24) | 0.49 |  | 0.22 | (-0.14, 0.57) | 0.24 |
| Household Income |  |  |  |  |  |  |  |
| €2200 / month or less | ref | | |  | ref | | |
| More than €2200 / month | 0.06 | (-0.07, 0.18) | 0.37 |  | 0.03 | (-0.08, 0.15) | 0.59 |
|  |  |  |  |  |  |  |  |
| Maternal history of psychosis | -0.12 | (-0.56, 0.31) | 0.58 |  | 0.31 | (-0.05, 0.67) | 0.09 |
| Paternal history of psychosis | -0.02 | (-0.52, 0.48) | 0.95 |  | -0.05 | (-0.63, 0.52) | 0.85 |
|  |  |  |  |  |  |  |  |
| Maternal psychopathology symptoms | 0.01 | (-0.14, 0.16) | 0.92 |  | -0.10 | (-0.27, 0.06) | 0.23 |
| Paternal psychopathology symptoms | 0.03 | (-0.16, 0.21) | 0.76 |  | 0.02 | (-0.20, 0.24) | 0.85 |
|  |  |  |  |  |  |  |  |
| Maternal age at child's birth | 0.01 | (0.00, 0.02) | 0.07 |  | 0.00 | (-0.02, 0.01) | 0.59 |
| Paternal age at child's birth | 0.00 | (-0.01, 0.01) | 0.54 |  | 0.00 | (-0.01, 0.01) | 0.87 |
|  |  |  |  |  |  |  |  |
| Child in utero exposure to smoking | -0.06 | (-0.13, 0.01) | 0.09 |  | -0.04 | (-0.11, 0.03) | 0.30 |
|  |  |  |  |  |  |  |  |
| Intercept | -2.53 | (-3.51, -1.56) | < 0.01 |  | 2.23 | (1.19, 3.27) | < 0.01 |
|  |  |  |  |  |  |  |  |
| *a. All models use inverse probability of attrition weights to account for differential attrition from baseline.* | | | | | | | |
| *b. These "unweighted" global outcome measures weight each tract equally, i.e., they are the standardized arithmetic mean FA and MD values of all 27 tracts delineated by AutoPtx regardless of size.* | | | | | | | |
|  |  |  |  |  |  |  |  |

Section 8. Global axial diffusivity and global radial diffusivity.

| **Supplement Section 8. Associations between family functioning and two mean measures (unweighted and weighted by tract volume) of global axial diffusivity and global radial diffusivity in preadolescence. n = 2,747.** | | | | | | | | |
| --- | --- | --- | --- | --- | --- | --- | --- | --- |
|  |  | Global AD | | |  | Global RD | | |
|  |  | β | 95% CI | *p* |  | β | 95% CI | *p* |
| Prenatal |  |  |  |  |  |  |  |  |
| Min. adjusted | Unweighted | -0.02 | (-0.15, 0.10) | 0.690 |  | -0.15 | (-0.26, -0.04) | 0.008 |
| Min. adjusted | Weighted | -0.02 | (-0.14, 0.11) | 0.780 |  | -0.12 | (-0.23, -0.01) | 0.038 |
|  |  |  |  |  |  |  |  |  |
| Fully adjusted | Unweighted | -0.10 | (-0.22, 0.03) | 0.145 |  | -0.16 | (-0.27, -0.04) | 0.007 |
| Fully adjusted | Weighted | -0.05 | (-0.18, 0.08) | 0.429 |  | -0.12 | (-0.23, -0.01) | 0.040 |
|  |  |  |  |  |  |  |  |  |
| Mid-childhood, not adjusted for prenatal score | | | |  |  |  |  |  |
| Min. adjusted | Unweighted | -0.02 | (-0.14, 0.10) | 0.781 |  | 0.00 | (-0.11, 0.12) | 0.992 |
| Min. adjusted | Weighted | -0.03 | (-0.15, 0.09) | 0.652 |  | 0.01 | (-0.11, 0.12) | 0.926 |
|  |  |  |  |  |  |  |  |  |
| Fully adjusted | Unweighted | -0.06 | (-0.18, 0.06) | 0.333 |  | 0.01 | (-0.11, 0.12) | 0.931 |
| Fully adjusted | Weighted | -0.05 | (-0.18, 0.07) | 0.420 |  | 0.01 | (-0.11, 0.13) | 0.850 |
|  |  |  |  |  |  |  |  |  |
| Mid-childhood, adjusted for prenatal score | | | |  |  |  |  |  |
| Min. adjusted | Unweighted | -0.01 | (-0.14, 0.12) | 0.880 |  | 0.06 | (-0.06, 0.18) | 0.357 |
| Min. adjusted | Weighted | -0.02 | (-0.15, 0.10) | 0.703 |  | 0.05 | (-0.07, 0.17) | 0.414 |
|  |  |  |  |  |  |  |  |  |
| Fully adjusted | Unweighted | -0.04 | (-0.17, 0.09) | 0.560 |  | 0.05 | (-0.07, 0.17) | 0.419 |
| Fully adjusted | Weighted | -0.04 | (-0.17, 0.09) | 0.537 |  | 0.05 | (-0.08, 0.17) | 0.460 |
|  |  |  |  |  |  |  |  |  |
| *a. "Unweighted" global measures weight each tract equally, i.e., they are the standardized arithmetic mean AD and RD values of all 27 tracts delineated by AutoPtx regardless of size. "Weighted" measures are weighted by tract volume.* | | | | | | | | |
| *b. Minimally adjusted models include covariates for child age at scan, sex, and ethnicity.* | | | | | | | | |
| *c. Fully adjusted models account for child age at scan, sex, ethnicity, household income, highest level of parental education, maternal and paternal history of psychosis, prenatal maternal and paternal psychopathology symptoms (for prenatal models), early-childhood maternal and paternal psychopathology symptoms (for mid-childhood models), maternal and paternal age at child's birth, and child in utero exposure to smoking.* | | | | | | | | |
| *d. All models use inverse probability of attrition weights to account for differential attrition from baseline.* | | | | | | | | |
|  | | | | | | | | |

Section 9. Prenatal functioning and global FA and MD – spline model results.

| **Supplement Section 9. Piecewise continuous linear spline model results for the association between prenatal family functioning, unweighted global MD, and unweighted global FA.** | | | | | | | | | | | | |
| --- | --- | --- | --- | --- | --- | --- | --- | --- | --- | --- | --- | --- |
|  | Global FA | | | | |  | Global MD | | | | |  |
|  | Pre-knot slope | |  | Post-knot slope | |  | Pre-knot slope | |  | Post-knot slope | |  |
| Knot | β | 95% CI |  | β | 95% CI |  | β | 95% CI |  | β | 95% CI |  |
| None | 0.11 | (0.00, 0.21) |  |  |  |  | -0.15 | (-0.28, -0.03) |  |  |  |  |
| 3.0 | 0.16 | (-0.13, 0.44) |  | 0.08 | (-0.06, 0.23) |  | -0.25 | (-0.62, 0.13) |  | -0.12 | (-0.26, 0.03) |  |
| 2.9 | 0.16 | (-0.17, 0.48) |  | 0.09 | (-0.05, 0.23) |  | -0.27 | (-0.71, 0.17) |  | -0.12 | (-0.26, 0.02) |  |
| 2.8 | 0.18 | (-0.19, 0.55) |  | 0.09 | (-0.05, 0.22) |  | -0.29 | (-0.80, 0.22) |  | -0.12 | (-0.26, 0.01) |  |
| 2.7 | 0.24 | (-0.18, 0.66) |  | 0.08 | (-0.05, 0.21) |  | -0.31 | (-0.91, 0.28) |  | -0.13 | (-0.26, 0.00) |  |
| 2.6 | 0.35 | (-0.12, 0.83) |  | 0.07 | (-0.05, 0.20) |  | -0.41 | (-1.10, 0.28) |  | -0.12 | (-0.25, 0.00) |  |
| 2.5 | 0.50 | (-0.04, 1.04) |  | 0.07 | (-0.05, 0.19) |  | -0.55 | (-1.34, 0.24) |  | -0.12 | (-0.24, 0.00) |  |
|  |  |  |  |  |  |  |  |  |  |  |  |  |
| *1. Fully adjusted models account for child age at scan, biological sex, ethnicity, household income, highest level of parental education achieved, maternal and partner history of psychosis, maternal and partner psychopathology symptoms, maternal and paternal age at child's birth, and child in utero exposure to smoking.* | | | | | | | | | | | | |
| *2. The post-knot β is the estimated absolute slope, i.e., not merely the change in slope relative to the pre-knot β.* | | | | | | | | | | | | |
| *3. Global outcomes are unweighted by tract volume. Results from tract-volume weighted outcomes were similar and thus omitted.* | | | | | | | | | | | | |

Section 10.

| **Supplement Section 10. Associations between family functioning and two mean measures (unweighted and weighted by tract volume) of global fractional anisotropy and global mean diffusivity in preadolescence in a subsample of Dutch / other European participants. Fully adjusted models only.**  **n = 2,727.** | | | | | | | | |
| --- | --- | --- | --- | --- | --- | --- | --- | --- |
|  |  | Global FA | |  |  | Global MD | |  |
|  |  | β | 95% CI | *p* |  | β | 95% CI | *p* |
| Prenatal |  |  |  |  |  |  |  |  |
| Subsample:  Dutch / Euro. | Unweighted | 0.14 | (0.01, 0.28) | 0.037 |  | -0.15 | (-0.32, 0.03) | 0.097 |
|  | Weighted | 0.13 | (-0.00, 0.27) | 0.058 |  | -0.11 | (-0.28, 0.06) | 0.213 |
|  |  |  |  |  |  |  |  |  |
| Full sample | Unweighted | 0.11 | (0.00, 0.21) | 0.049 |  | -0.15 | (-0.28, -0.03) | 0.014 |
|  | Weighted | 0.12 | (0.01, 0.23) | 0.040 |  | -0.10 | (-0.23, 0.02) | 0.091 |
|  |  |  |  |  |  |  |  |  |
| Mid-childhood, not adjusted for prenatal score | | | | |  |  |  |  |
| Subsample:  Dutch / Euro. | Unweighted | 0.03 | (-0.11, 0.17) | 0.665 |  | -0.02 | (-0.17, 0.13) | 0.775 |
|  | Weighted | 0.05 | (-0.09, 0.19) | 0.491 |  | 0.00 | (-0.14, 0.14) | 0.986 |
|  |  |  |  |  |  |  |  |  |
| Full sample | Unweighted | -0.04 | (-0.16, 0.08) | 0.500 |  | -0.02 | (-0.14, 0.10) | 0.715 |
|  | Weighted | -0.04 | (-0.16, 0.08) | 0.515 |  | -0.01 | (-0.13, 0.11) | 0.826 |
|  |  |  |  |  |  |  |  |  |
| Mid-childhood, adjusted for prenatal score | | | | |  |  |  |  |
| Subsample:  Dutch / Euro. | Unweighted | -0.01 | (-0.16, 0.14) | 0.900 |  | 0.02 | (-0.13, 0.17) | 0.773 |
|  | Weighted | 0.01 | (-0.13, 0.16) | 0.856 |  | 0.03 | (-0.11, 0.18) | 0.655 |
|  |  |  |  |  |  |  |  |  |
| Full sample | Unweighted | -0.07 | (-0.20, 0.05) | 0.247 |  | 0.02 | (-0.10, 0.14) | 0.748 |
|  | Weighted | -0.08 | (-0.20, 0.05) | 0.235 |  | 0.02 | (-0.11, 0.14) | 0.809 |
|  |  |  |  |  |  |  |  |  |
| *a. "Unweighted" global measures weight each tract equally, i.e., they are the standardized arithmetic mean FA and MD values of all 27 tracts delineated by AutoPtx regardless of size. "Weighted" measures are weighted by tract volume.* | | | | | | | | |
| *b. "Full sample" estimates, reported in the main text Table 2, are provided above for comparison.* | | | | | | | | |
| *c. All models are fully adjusted and include covariates for child age at scan, sex, country of origin, household income, highest level of parental education, maternal and paternal history of psychosis, prenatal maternal and paternal psychopathology symptoms (for prenatal models), early-childhood maternal and paternal psychopathology symptoms (for mid-childhood models), maternal and paternal age at child's birth, and child in utero exposure to smoking.* | | | | | | | | |
| *d. All models use inverse probability of attrition weights to account for differential attrition from baseline.* | | | | | | | | |
|  |  |  |  |  |  |  |  |  |

Section 11.

Generation R Study researchers enrolled participants from Rotterdam’s diverse population of families originating from many different countries, and as such, they asked parents about their social identities, which included information on both the participants’ and the participants’ parents’ countries of birth.

Using this data, the Generation R Study defined and operationalized “ethnicity” according to the official classification scheme used by Statistics Netherlands, the national statistics body of the Netherlands (Kooijman et al., 2016). Mothers and fathers born in the Netherlands to parents who were also born in the Netherlands were classified as Dutch. If one of the participant’s parents was born outside of the Netherlands, the participant was classified as having a “non-Dutch ethnic origin.” If both parents were born outside the Netherlands, the participant’s ethnicity was determined based on the participant’s mother’s country of birth. In turn, each participating child’s ethnicity was defined based on the ethnicities of his or her parents.

Given the diversity of Rotterdam and its surrounding area, participants reported a wide spectrum of ethnicities, including Dutch, Moroccan, Turkish, Dutch Antillean, Surinamese, Cape Verdean, and several other non-Dutch European, Asian, African, North American, and South American ethnicities. Because many of these “ethnicities” have a small number of participants in the Generation R Study, we operationalized this data following prior work in the Generation R Study as a five-category variable we refer to as “ethnicity.” Categories include European (non-Turkish), Turkish, Moroccan, Surinamese, and Other Ethnicity / National Origin.

Section 12.

| **Supplement Section 12. Associations between prenatal family functioning and preadolescent global fractional anisotropy and global mean diffusivity (weighted and unweighted by tract volume) and CBCL total behavior problem score. n = 2,727.** | | | |
| --- | --- | --- | --- |
|  |  |  |  |
|  | CBCL Total Score | |  |
|  | β | 95% CI | *p* |
| Prenatal family functioning | -3.37 | (-5.48, -1.26) | < 0.01 |
|  |  |  |  |
| Global FA |  |  |  |
| Unweighted | 0.24 | (-0.57, 1.05) | 0.56 |
| Weighted | -0.02 | (-0.83, 0.79) | 0.95 |
|  |  |  |  |
| Global MD |  |  |  |
| Unweighted | -0.02 | (-0.86, 0.82) | 0.97 |
| Weighted | 0.08 | (-0.73, 0.88) | 0.85 |
| *a. "Unweighted" global measures weight each tract equally, i.e., they are the standardized arithmetic mean FA and MD values of all 27 tracts delineated by AutoPtx regardless of size. "Weighted" measures are weighted by tract volume.* | | | |
| *b. All models are fully adjusted with covariates for child age at scan, sex, ethnicity, household income, highest level of parental education, maternal and paternal history of psychosis, prenatal maternal and paternal psychopathology symptoms (for prenatal models), early-childhood maternal and paternal psychopathology symptoms (for mid-childhood models), maternal and paternal age at child's birth, and child in utero exposure to smoking.* | | | |
| *c. All models use inverse probability of attrition weights to account for differential attrition from baseline.* | | | |
|  |  |  |  |

References

de Groot, M., Vernooij, M. W., Klein, S., Ikram, M. A., Vos, F. M., Smith, S. M., Niessen, W. J., & Andersson, J. L. R. (2013). Improving alignment in Tract-based spatial statistics: Evaluation and optimization of image registration. *NeuroImage*, *76*, 400–411. https://doi.org/10.1016/j.neuroimage.2013.03.015

Kooijman, M. N., Kruithof, C. J., van Duijn, C. M., Duijts, L., Franco, O. H., van IJzendoorn, M. H., de Jongste, J. C., Klaver, C. C. W., van der Lugt, A., Mackenbach, J. P., Moll, H. A., Peeters, R. P., Raat, H., Rings, E. H. H. M., Rivadeneira, F., van der Schroeff, M. P., Steegers, E. A. P., Tiemeier, H., Uitterlinden, A. G., … Jaddoe, V. W. V. (2016). The Generation R Study: Design and cohort update 2017. *European Journal of Epidemiology*, *31*(12), 1243–1264. https://doi.org/10.1007/s10654-016-0224-9

Rubin, D. B. (1996). Multiple Imputation After 18+ Years. *Journal of the American Statistical Association*, *91*(434), 473–489. JSTOR. https://doi.org/10.2307/2291635

White, T., Muetzel, R. L., El Marroun, H., Blanken, L. M. E., Jansen, P., Bolhuis, K., Kocevska, D., Mous, S. E., Mulder, R., Jaddoe, V. W. V., van der Lugt, A., Verhulst, F. C., & Tiemeier, H. (2018). Paediatric population neuroimaging and the Generation R Study: The second wave. *European Journal of Epidemiology*, *33*(1), 99–125. https://doi.org/10.1007/s10654-017-0319-y
